# The CIRCULAR model: advancing public health through citizen science initiated clinical and mechanistic research in atrial fibrillation

**DOI:** 10.3389/fpubh.2026.1735912

**Published:** 2026-04-13

**Authors:** Myrthe F. Kuipers, Maurice L. Remy, Amélie C. T. Collinet, Kennedy Silva Ramos, Carina A. C. M. Pittens, Michiel van Oudheusden, Ronja Laurila, Jan van der Werke, Elza D. van Deel, Umut Konus, Natasja M. S. de Groot, Bianca J. J. M. Brundel

**Affiliations:** 1 Department of Marketing, Economics and Business Administration, Amsterdam Business School, University of Amsterdam, Amsterdam, Netherlands; 2 Athena Institute, VU Amsterdam, Amsterdam, Netherlands; 3 Department of Physiology, Amsterdam UMC, Location Vrije Universiteit, Amsterdam Cardiovascular Sciences, Heart Failure and Arrhythmias, Amsterdam, Netherlands; 4 Atrial Fibrillation Innovation Platform Foundation, Amsterdam, Netherlands; 5 Department of Cardiology, Erasmus Medical Center, Rotterdam, Netherlands; 6 Microelectronics, Signal Processing Systems, Faculty of Electrical Engineering, Mathematics and Computer Sciences, Delft University of Technology, Delft, Netherlands

**Keywords:** atrial fibrillation, citizen-science, co-creation, patient foundation, patient involvement

## Abstract

Citizen science is a transformative approach to advancing health research by bridging the gap between researchers and the public. The Dutch CIRCULAR model, which addresses atrial fibrillation (AF), the most common cardiac arrhythmia worldwide, offers a unique example of applying citizen science in biomedical and public health research. The project was established to systematically include people with AF and their families as partners in research, ensuring that their lived experiences inform priorities, study designs, and interventions. A central role is played by the online health community of the AFIP foundation, which engages the AF community through blog articles, forums, social media, and outreach campaigns. These activities stimulate dialogue, enhance health literacy, empower individuals to contribute hypotheses and solutions, and function as a marketing strategy to attract and retain diverse participants. By sharing outcomes through open-access formats and direct communication with participants, CIRCULAR creates a feedback loop between citizens and researchers that fuels new research directions in AF. Early activities have demonstrated the value of this approach. Patient-reported triggers and suppressors of AF episodes, including psychological stress and lifestyle factors, informed laboratory investigations and led to the co-design of clinical interventions such as dietary programs. These examples illustrate how co-creation can shape both preclinical and clinical research as well as citizen and student education. This review discusses how citizen science is conceptualized and implemented in the CIRCULAR model, presents ongoing and future activities, and reflects on the added value of patient involvement for public health and biomedical innovation. By embedding citizens throughout the research process, and actively engaging them through targeted outreach, CIRCULAR advances patient-centered innovation, strengthens empowerment and health literacy, and provides lessons for future participatory initiatives in complex chronic disease research.

## 1 Introduction

Over the past decade, the health information landscape has undergone a profound transformation. Where individuals once primarily relied on physicians and other healthcare professionals for medical advice, today’s patients increasingly act as informed health consumers navigating a diverse ecosystem of information sources. Emerging health actors, such as AI-driven tools, medical technology companies, and patient influencers, have reshaped how people access and interpret health information (1). As a result, individuals now turn to digital environments not only to seek guidance about medical conditions but also to connect with others who share similar experiences.

One impactful development within this evolving landscape is the rise of online health communities (OHCs). These virtual spaces facilitate interactions between patients, caregivers, clinicians, and other stakeholders across the healthcare ecosystem (2). OHCs function as hubs for peer-to-peer exchange, where members share practical advice, emotional support, and experiential knowledge that often extend beyond the scope of traditional medical consultations (3). Studies have shown that such online social support can contribute to positive health outcomes, including improved medication adherence (4), successful weight management (5), reduced smoking (6), and ultimately lower mortality rates (7). Importantly, OHCs not only benefit patients, they also provide clinicians and researchers with access to patient perspectives, unreported symptoms, and real-world experiences that may inform and improve clinical practice and policy development (2).

Within this context, patient platforms have shown particular promise in chronic disease management, where daily experiences play a critical role in treatment success and quality of life. A notable example is the AFIP Foundation, which employs citizen science (CS) to advance atrial fibrillation (AF) research by enhancing collaboration, co-creation, and shared data generation between researchers and the public with the aim of personalizing AF treatment. AF is the most common sustained cardiac arrhythmia worldwide, affecting more than 60 million people globally and projected to reach 14–17 million cases in Europe by 2030 (8, 9). Unlike many cardiometabolic diseases that may progress silently, AF is often highly symptomatic. Palpitations, fatigue, shortness of breath, and anxiety drive many patients to seek care, yet these episodes are often triggered by modifiable factors such as psychological stress, alcohol intake, diet, allergies, or environmental exposures (10–12). Current AF care pathways, however, remain fragmented and suboptimal, with limited integration of patient experience. Despite advances in pharmacological and interventional therapies, including ablation, existing treatments are largely treating symptoms and fail to halt disease progression. Up to 85% of patients on pharmacological treatment and nearly 50% of those undergoing ablation continue to experience symptoms (10). The condition is highly heterogeneous, episodic, and strongly influenced by lifestyle, psychosocial, and environmental factors that are often difficult to capture in controlled clinical settings. As a result, individuals living with AF frequently develop detailed experiential knowledge about personal triggers, suppressors, and self-management strategies over time. Yet this patient experience has rarely been integrated into formal research design or policy development. As a result, potentially valuable patient insights remain untapped, and innovation pathways risk becoming misaligned with the needs and realities of those most affected. These circumstances make AF particularly well suited for CS approaches in which lived experience functions as a source of hypotheses that can be prioritized and subsequently tested in preclinical and clinical research.

CS offers a valuable mechanism for integrating insights from individuals with lived experience of a particular health condition into new research and guideline development. This approach enables researchers to obtain novel, firsthand insights into specific phenomena and to address both scientific and societal challenges (13). According to Haag et al. (14) CS refers to “the active engagement and collaboration with the public (i.e., ‘citizens’) in scientific endeavors”. Within the health domain, this framework seeks to reposition patients as co-creators of knowledge, where their experiential understanding and personal perspectives shape not only what is studied but also how findings are interpreted and applied (15). To bridge the gap in the field of AF, the Dutch CIRCULAR consortium was established, building on the AFIP Foundation’s central OHC. The consortium applies CS in preclinical and clinical research in cardiology, which are fields in which CS has rarely been implemented. Through the OHC of the AFIP Foundation, CIRCULAR actively engages patients diagnosed with AF and their families via blogs, forums, social media, and outreach campaigns, fostering continuous dialogue and collaboration between citizens and researchers. These activities not only promote participation, but also strengthen health literacy, empower individuals, and create an ongoing dialogue between citizens and researchers (16, 17). By systematically incorporating lived experiences into hypothesis generation, laboratory investigations, and intervention design, CIRCULAR seeks to connect the realities of AF patients with the biomedical research pipeline. By capturing how patients and caregivers experience AF care, including inconsistencies, gaps, and inequities, CS can help to develop more personalized and equitable treatment pathways. Insights from diverse populations can inform interventions, improve the consistency of clinical guidelines, and ensure that recommendations better reflect the realities of everyday AF care.

Despite growing recognition of CS as a valuable approach in health research, existing applications have largely focused on agenda setting, data collection, implementation research, and service improvement in public health and health policy domains, with comparatively limited integration into preclinical and mechanistic biomedical research (18, 19). In much of the current CS literature, citizen contributions inform *what* is studied or *how* interventions are implemented, but rarely shape the generation and experimental testing of mechanistic hypotheses at the molecular, cellular, or physiological level. This review advances the field by explicitly addressing this gap. By positioning lived experience as a starting point for hypothesis generation, prioritization, and subsequent investigation in human-based laboratory models and translational studies, the CIRCULAR model extends citizen participation into stages of the biomedical research pipeline where CS remains underdeveloped. The novelty of this contribution lies not in introducing CS as a participatory principle per se, but in demonstrating how citizen-derived insights can function as a scientifically generative input across preclinical, clinical, and public health domains, thereby bridging experiential knowledge and mechanistic biomedical innovation in a structured manner.

This framework-advancing review presents the conceptualization and implementation of CS in the CIRCULAR project, highlights early outcomes, and reflects on both the opportunities and challenges of embedding patient engagement in (bio)medical and public health innovation. In doing so, the present article discusses how CS can contribute to advancing patient-centered approaches to complex chronic diseases such as AF, and provides lessons for scaling citizen participation across the health research continuum. First, this paper outlines the origins of CS and the development of the CIRCULAR model. Next, it introduces the core pillars of CIRCULAR and illustrates their practical implementation within the health research and educational context. Finally, the paper discusses the proposed framework and reflects on its broader implications for other areas within the healthcare domain.

## 2 History of citizen science and project CIRCULAR: origins and rationale

### 2.1 Citizen science in health research: conceptual background

The term CS has two distinct origins, which have shaped its varied definitions and meanings in both academic and non-academic contexts. In the 1990s, Irwin (20) and Bonney (21) independently coined the term from different perspectives. Irwin conceptualized CS as an approach, in which citizens actively develop and enact knowledge production to meet their own societal needs. In contrast, Bonney used the term to describe the contributory work of volunteers supporting professional scientists, as in his case of citizens collecting ornithological data. Building on these two traditions, Cooper and Lewenstein (22) later proposed a hybrid understanding of CS that integrates both the contributory and democratic dimensions: “a gold standard for CS practice in which people do more than contribute data, and researchers do more than use the data”. Similarly, the European Citizen Science Association (ECSA, 2015) defined CS as the active involvement of citizens in scientific endeavors that generate new knowledge or understanding, where citizens can act as contributors, collaborators, or even project leaders (23).

### 2.2 Citizen science in the health domain

Within the health research context, CS has been promoted as a means to democratize the research system, aligning closely with Irwin’s participatory vision (20, 24). The argument for citizen involvement in health research is normative, epistemic, and instrumental. From a normative perspective, citizens have a legitimate right to participate in shaping research agendas, particularly in health, where research outcomes directly influence individual and societal wellbeing (24, 25). Since health research is often publicly funded, and its results affect public welfare, it is reasonable to argue that citizens should play an active role in determining what constitutes health priorities and acceptable care. In line with the right to health articulated in international frameworks such as the Universal Declaration of Human Rights, involvement of citizens can thus be understood as part of realizing this fundamental right. From an epistemic perspective, involving patients and citizens enhances the quality, and relevance of research. Citizen participation introduces experiential and contextual knowledge that complements professional expertise, leading to outcomes that are better aligned with real-world needs (26–29). The use of CS is also seen as instrumentally valuable by increasing legitimacy of research. Empirical evidence suggests that participatory research not only improves data relevance and methodological validity but also increases public trust and the likelihood of successful implementation of research outputs (30, 31). Depending on the context of implementing CS, e.g., by whom or with which objective, one of the rationales might be dominant. This can result in different forms of CS, that range from contributory approaches rooted in Bonney’s model to democratic approaches grounded in Irwin’s. All forms of CS can be relevant to contemporary health research, particularly where patients and citizens engage with issues that directly affect their lives, such as the case of AF.

### 2.3 Integrating citizen science and patient involvement

In health research, the concept of patient involvement (PI) is closely related to CS, and the two are increasingly overlapping in practice (32). PI, like CS, lacks a universal definition but varies across disciplines and traditions (33). In this paper, and in the CIRCULAR consortium, we use the definition by Schölvinck (34), who describes PI as “the involvement of patients (or their representatives) in health research decision-making on the basis of their experiential knowledge, thereby enhancing the legitimacy and rationality of the decision-making process and encouraging a mutual learning process between stakeholders”. This conceptual proximity makes it useful to draw on insights from both literatures. PI extends the evidence base for best practices in participation and provides concrete frameworks for co-design, co-creation, and shared decision-making. While CS emphasizes inclusivity and open participation, PI highlights the depth of experiential expertise patients bring to research processes. In practice, the boundaries between the two concepts are often overlapping; citizens participating in health research are frequently patients, and both methodologies employ similar participatory tools and frameworks.

### 2.4 The need for further implementation in biomedical research

Despite increasing recognition of CS as a valuable concept in health research, implementation remains uneven, and challenges persist (28, 35–38). Key barriers include a lack of institutional incentives, uneven data quality standards, and a limited understanding of how to embed participatory approaches into complex biomedical research workflows (13, 39, 40). These challenges are particularly evident in preclinical and laboratory settings, where participatory models are underdeveloped and empirical evidence remains scarce (18, 19). To advance this field, more initiatives are needed that both implement and critically analyze CS practices across the full research pipeline, from idea generation to translational testing. The CIRCULAR project is one such initiative, pioneering the integration of CS into biomedical, preclinical, clinical and public health research in cardiology. By systematically embedding citizen participation into the study of AF, CIRCULAR provides a unique opportunity to examine how experiential knowledge can generate hypotheses, guide mechanistic research, and accelerate innovation toward personalized care.

### 2.5 The CIRCULAR project

The CIRCULAR consortium was established in 2022 in the Netherlands with funding from the Dutch NWA-ORC program (41). It builds on the citizen collaboration model of the AFIP Foundation, an independent nonprofit founded in 2016 to strengthen collaboration between citizens living with AF, researchers and health professionals. Whereas AF research has traditionally been dominated by clinical and biomedical models with limited integration of patient experience, CIRCULAR’s mission is to systematically collect citizen insights and integrate them into multidisciplinary approaches, bringing together academic researchers, hospitals, universities of applied sciences, companies, and nonprofit organizations. This makes CIRCULAR one of the first initiatives worldwide to embed CS into preclinical biomedical research in the field of cardiology.

The Dutch AFIP Foundation functions as the main entry point for citizen participation in CIRCULAR. Its online health community currently attracts nearly 12,000 organic monthly visitors. Members could learn about AF through academically grounded content translated into accessible language, and they have the opportunity to engage in peer exchange on symptoms, triggers, treatment responses, and lifestyle strategies. This interactive environment is supposed to foster health literacy, empower patients to self-manage their condition, and create a two-way channel where researchers can identify promising patient-reported patterns. A testimonial from one member, who reported disappearance of AF episodes after antihistamine intake, demonstrates how incidental experiences can generate hypotheses for further research: “*My motivation to continue sharing my experiences with the community mainly stems from the fact that you, the AFIP foundation, have taken my incidental finding […] seriously.*” While anecdotal, such experiences are currently investigated on the mechanistic level in the preclinical setting within project CIRCULAR.

## 3 Building the AFIP community

**Figure 1:**
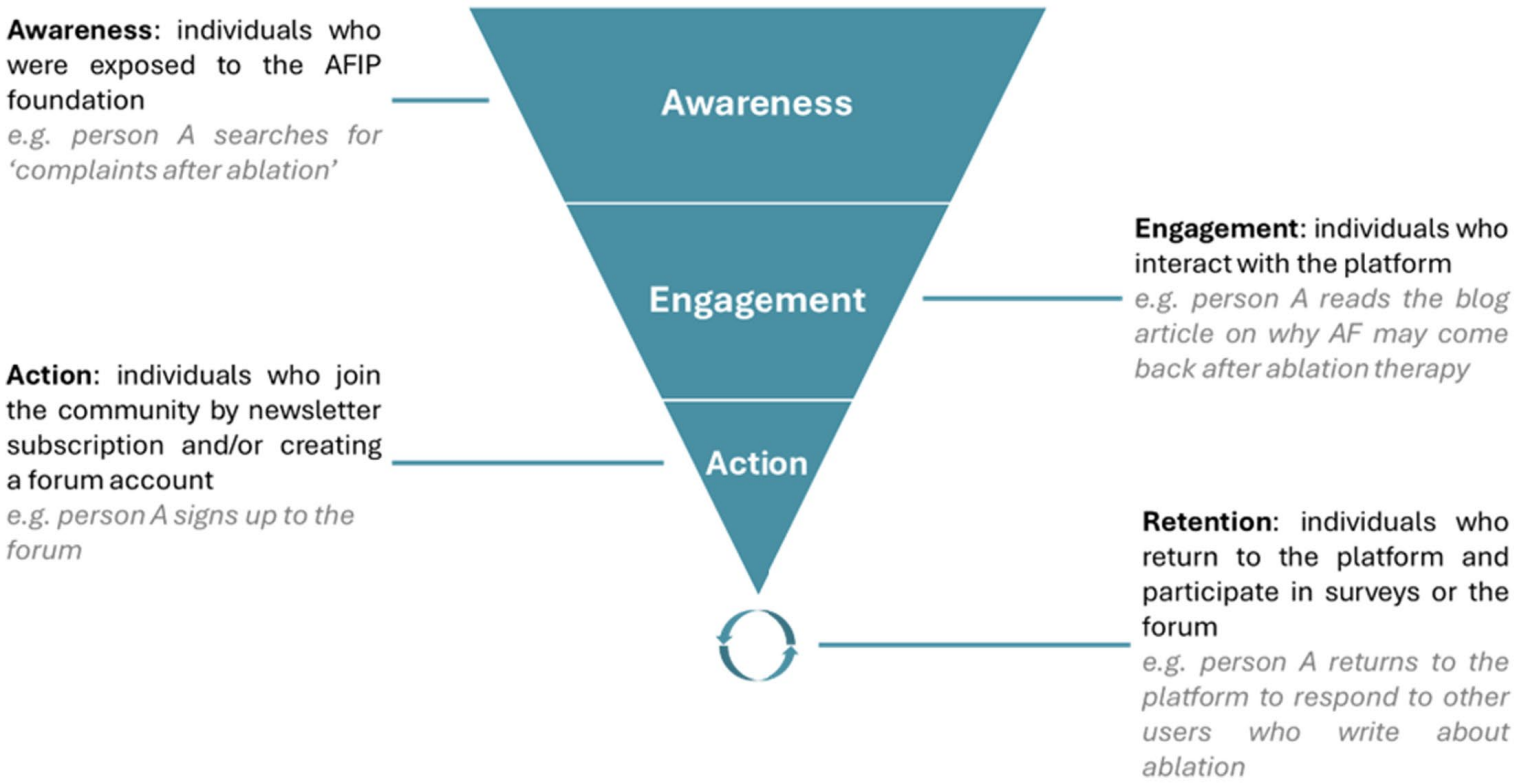
The AF journey: marketing funnel model for building the AFIP Foundation community. Illustration of the four-phase AF journey funnel (awareness, engagement, action, and retention) applied by the AFIP Foundation to attract and sustain participation among individuals with AF. Adapted from the classical AIDA marketing model, in the awareness phase, public outreach through social media and online campaigns introduces AFIP as a trustworthy source of AF information. The engagement phase deepens interaction through online forums, newsletters, and educational content that stimulate dialogue between citizens and researchers. In the action phase, community members subscribe to the newsletter and actively contribute by completing surveys, sharing personal experiences, or participating in research initiatives. Finally, the retention phase focuses on sustaining involvement through feedback on research results, community updates, and continued opportunities for participation.

As previously discussed, the AFIP Foundation was established in the Netherlands to serve as a platform where individuals with AF can access reliable information about the condition, recognize warning signs, and share self-tracked insights within the community. The foundation forms the cornerstone of the CIRCULAR consortium and was initiated without a pre-existing community of AF patients. To attract website visitors, the foundation implemented online health marketing. Online health marketing campaigns represent an increasingly important mechanism for disseminating health information and engaging diverse population groups, including underserved, minority, and segmented audiences, particularly in the context of chronic and non-communicable diseases. By the implementation of tailored messaging and targeted digital strategies, such campaigns can facilitate access to health-related information, tools, and services that may otherwise remain underutilized. Prior research demonstrates that marketing-based modeling approaches can be instrumental in addressing structural barriers to healthcare access, for example by evaluating alternative delivery strategies to sustain service provision in settings affected by physician shortages (42). Similarly, research conducted within Aboriginal communities illustrates how marketing strategies can contribute to decolonized healthcare delivery by aligning health interventions with the cultural, social, and institutional contexts of marginalized populations (43). These findings suggest that health marketing approaches have the potential to enhance health awareness, strengthen engagement, and support the adoption of online health services. The AFIP foundation utilized a marketing funnel, referred to as the “AF journey,” comprising four phases: awareness, engagement, action, and retention. This framework is adapted from the well-known Attention, Interest, Desire, Action (AIDA) stimulus–response model (44), with the “desire” phase omitted and “retention” added. While widely applied in marketing, this model was leveraged to grow the non-profit AF community. Figure 1 displays the AF journey funnel applied by the AFIP foundation. Moreover, the supplementary data file provides a thorough overview of this chapter with more detailed statistics, figures, and tables.

### 3.1 Generating awareness

**Figure 2:**
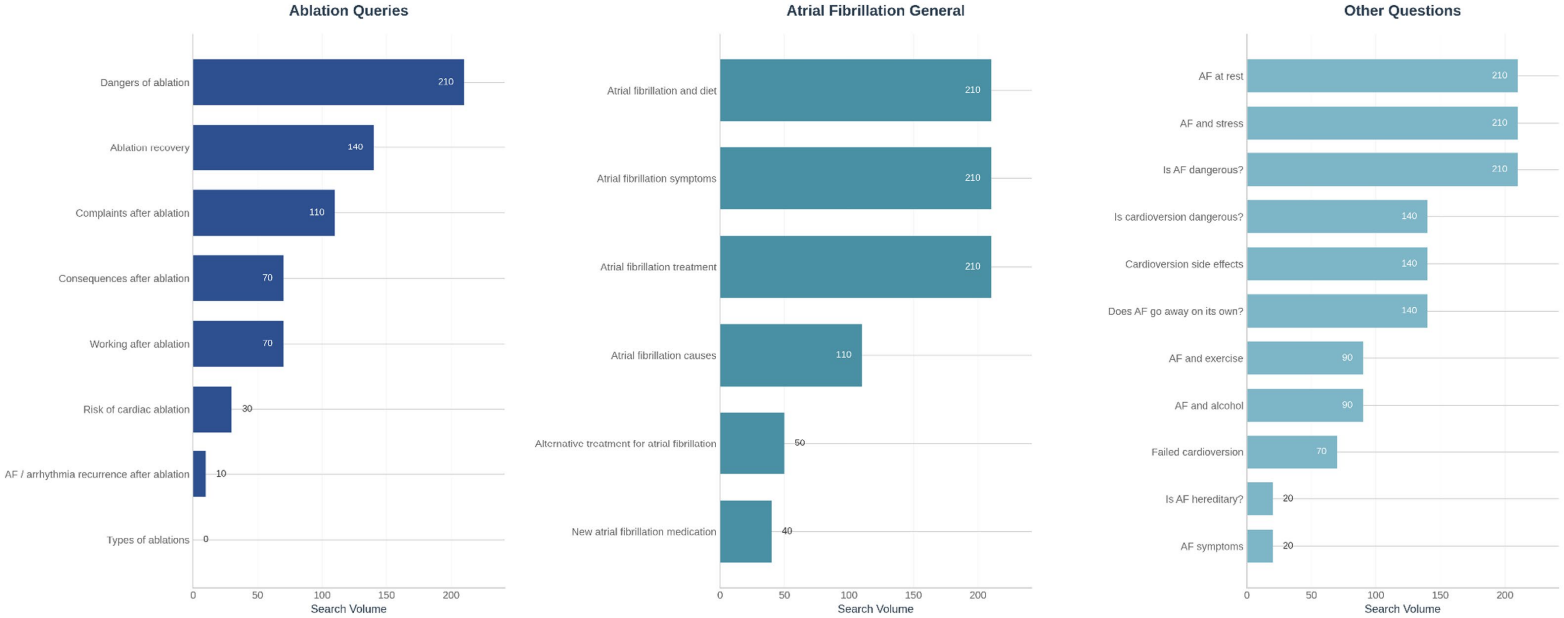
An overview of the more detailed keywords within the ablation, general AF, and ‘other’ AF segment for the month February in 2019. Keywords were translated from Dutch into English. The results indicate that individuals were searching for terms such as “complaints after ablation,” highlighting that many still experienced AFib symptoms post-treatment. Searches in the AF general and other categories reveal interest in the relationship between triggers and suppressors, including queries like “atrial fibrillation and diet” and “AF and stress.” These insights informed AFIP’s content strategy, guiding the creation of material tailored to these audience interests.

To stimulate exposure and raise awareness of the AFIP Foundation, online search behavior analyses were regularly conducted to identify the topics most frequently sought by individuals. The AFIP Foundation employed an organic inbound approach, designing website pages based on the most frequently searched topics in the Netherlands. Notably, many searches were related to triggers for AF (e.g., AF and stress), lifestyle habits (e.g., ‘Atrial fibrillation and diet’) or treatments (e.g., ‘Complaints after ablation’) (Figure 2). In response, the AFIP Foundation developed evidence-based informational content addressing common search engine queries, such as catheter ablation and the risk of AF recurrence, informed by prior research (45).

**Figure 3:**
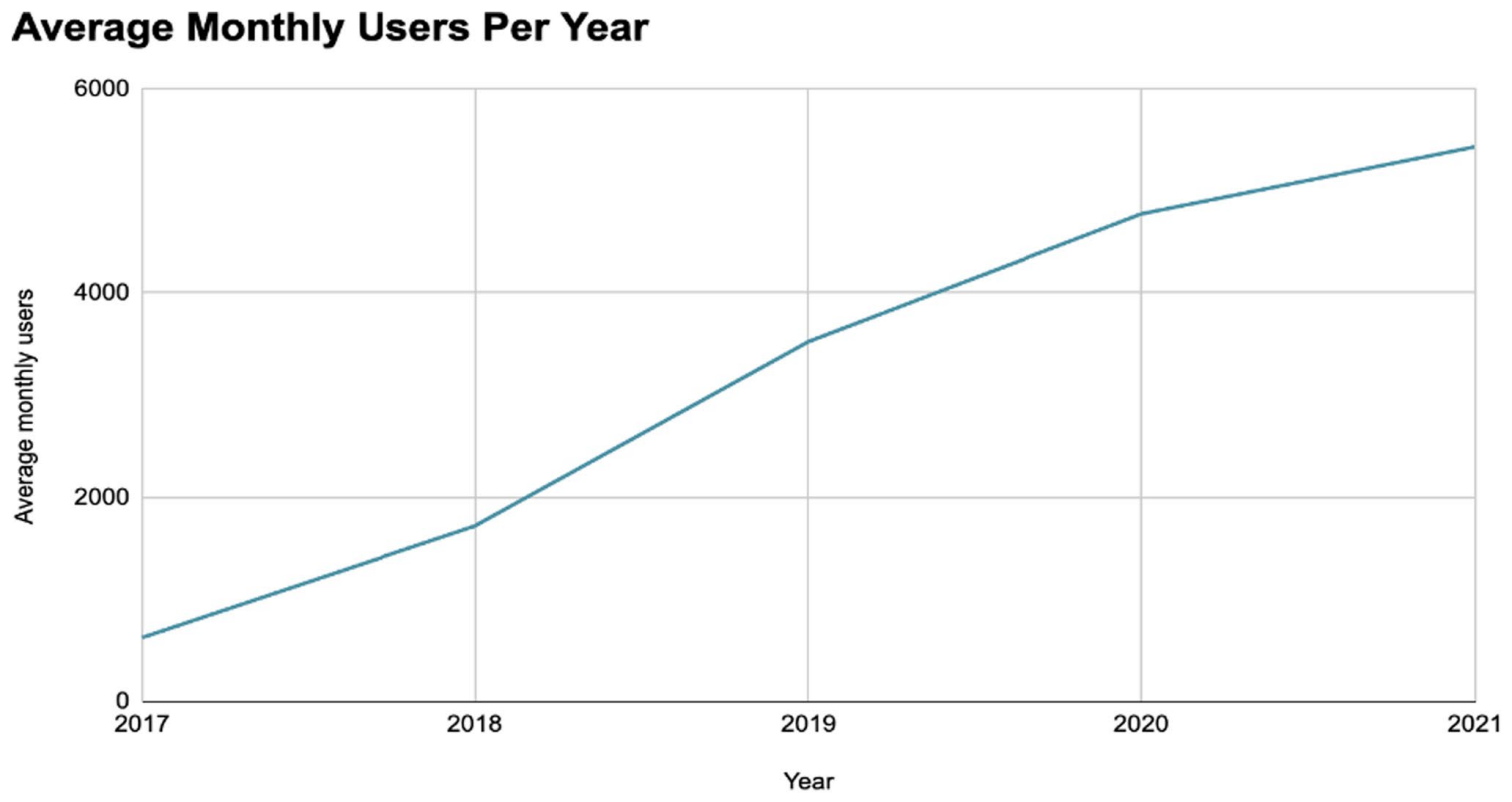
Overview of all users to the AFIP foundation website from May 1, 2016, to May 31, 2021 as measured by Google Universal Analytics (UA). Search engine optimization (SEO) is a long-term strategy, with effects becoming more apparent after content targeting user interests was introduced in early 2019. Within 2 years, the number of visitors more than doubled.

Building an audience through this approach requires time, as search engine optimization (SEO) is inherently a long-term strategy. Figure 3 demonstrates how this method grew the AFIP foundation’s user base from 300 monthly users at the start of 2017 to over 5,000 users after 4 years, ultimately exceeding 11,000 monthly users by the end of 2025. By offering accessible and evidence-based information, this approach enhances health literacy and raises awareness of CS initiatives among affected individuals. Awareness is a critical prerequisite for meaningful engagement in CS.

### 3.2 Generating engagement

The next step after generating traffic (awareness), is to make sure individuals stay engaged with the AFIP foundation. Within the marketing literature, there are multiple definitions of engagement. Examples are post replying behavior (46), sum of likes, shares, and comments (47), or eye-tracking to measure attentional focus (48). The measurement of engagement may be determined by the organization. Within the AFIP Foundation, engagement is assessed using metrics such as average session duration, requests for factsheets, contributions to forum discussions, attendance at physical events, and participation in brainstorming sessions.

Experimentation is critical to identify which communication strategies most effectively stimulate user engagement. As such, the AFIP Foundation, as part of the CIRCULAR consortium, tested 12 combinations of social media communication elements (i.e., emotion, patient-generated topics, narrative appeal, and linguistic style) on awareness (i.e., click-through rate) and engagement (i.e., session time, community subscriptions) (49). The campaign, conducted on Facebook and Instagram in the Netherlands, reached 795,812 users, with 18,426 visiting the platform. Fear with self-protection themes increased awareness, while love with affiliation or kin care enhanced engagement; expert narratives drove awareness, and patient stories drove engagement.

### 3.3 Stimulating action

Becoming a member represents the primary action objective for the AFIP Foundation. Members constitute a valuable pool of participants who contribute to research development by regularly completing surveys on topics such as stress, supplement use, and allergies. Communication with members primarily occurs via email, with the current membership base totaling *n* = 1,945 (October 2025). Over the past 4 years, an average of 67% of subscribers open emails [over three times the wellness industry average (19.2%)] and 19.3% click embedded links, including survey or event invitations, corresponding to 28.9% of openers (50).

High action among AFIP members is important for the discovery of important triggers and suppressors of AF. Frequently discussed topics on the forum are systematically collected and transformed into surveys to obtain more detailed insights into specific observations. Table 1 provides an overview of survey responses. For example, many forum participants report that AF episodes are triggered during periods of stress. To investigate the types of stress experienced, the timing of these events, and the associated demographic and behavioral characteristics of users, the AFIP foundation, in collaboration with psychologists in the Netherlands, developed a dedicated survey. This survey has been completed 782 times and contributes to the development of academic studies on this topic.

**Table 1:** Overview of survey topics and responses received (October 27, 2020 – October 20, 2025).

Survey topic	Participants (count)
Stress	782
Cardioversion	509
COVID-19	359
Pesticides	269
Experiences with AF	269
L-Glutamine	86
Histamine and allergies	201

Survey responses serve as a foundation for developing new research directions tailored to individual needs by the CIRCULAR consortium.

### 3.4 Improving retention

Once someone with AF converted to becoming a member and contributing to the platform by sharing insights, the aim is to retain that user. Member loyalty is a crucial aspect to grow organizations and to establish brand identification and trust among users (51). The AFIP foundation includes several methods to provide value so that individuals are incentivized to keep sharing insights. One of the methods includes organizing an annual symposium (attracting nearly 300 visitors in person and 100 visitors online) to inform the AF patient community about the latest research on AF and updates on observations people made, while it additionally allows input from the community to healthcare professionals. By highlighting the value of sharing lived experiences for personalizing AF treatments, this event might function as a cornerstone for future patient-centered research activities. Another method is the release of in-between status reports from the surveys that participants filled out. This is supposed to keep the audience informed and give recognition for the experiences shared. Finally, the AFIP foundation hosts webinars and brainstorming sessions with the community to discuss topics in more detail and to answer questions from the community. Active involvement of individuals with AF enables the inclusion in the scientific context via data sharing and incorporation of lived experiences, providing foundation for CIRCULAR.

## 4 From patient insights to Dutch research agenda

Members of the AFIP foundation’s OHC constitute the core resource on which the CIRCULAR framework is built. Insights provided by individuals living with AF offered substantial value, guiding the development of new research pathways. Following prioritization, these pathways are investigated in the laboratory to explore mechanistic relationships between identified triggers or suppressors and AF. Findings deemed significant proceed to a clinical phase, during which novel treatment approaches are tested. Successful interventions are subsequently disseminated to guideline developers and communicated back to AFIP foundation members and the remaining AF population, completing the CIRCULAR process. This chapter provides an overview of the CIRCULAR framework and describes the sequential stages involved in generating personalized AF research and treatment pathways.

### 4.1 Prioritizing AF experiences for new research pathways

After implementation of the marketing framework, the collected insights, through that framework, are prioritized to determine which ones are investigated further within the next phases of the CIRCULAR consortium. The framework is based on the PIE Prioritization framework developed by Chris Goward at WiderFunnel, which is commonly used in the field of growth hacking (52, 53). The prioritization framework is a prioritization methodology designed to guide marketing and growth teams in determining the sequence in which to implement optimization initiatives. The framework evaluates opportunities based on three key criteria: (1) Potential (the expected impact of a change), (2) Importance (the opportunity in terms of business impact), and (3) Ease (the level of effort, resources, and complexity required for implementation). To align the framework with the objectives of the CIRCULAR consortium and the nature of insights derived from the AFIP Foundation, the Brundel–Kuipers Prioritization Matrix (BKPM) was developed to integrate CS findings into the academic research cycle across laboratory and clinical contexts. Specifically, the ‘potential’ criterion was redefined to reflect the extent of existing scientific evidence related to a trigger reported by AFIP members. Triggers supported by prior research suggesting a plausible link with AF were considered to have higher potential for further investigation. The ‘importance’ criterion was replaced by the frequency with which a particular trigger was reported by members, assuming that frequently reported triggers represent more prominent or impactful observations within the community. Finally, the ‘ease’ criterion was reinterpreted as ‘ease of implementation’, referring to the resources, personnel, and administrative effort required to investigate the trigger. All three criteria were assigned equal weight, and the mean of the three scores (each rated on a scale from 1 to 10) was used to generate a composite prioritization score. A higher score indicates a higher priority for further study within subsequent stages of the CIRCULAR model (Table 2).

**Table 2:** Illustration of the Brundel–Kuipers Prioritization Matrix (BKPM), which ranks CS, reported triggers for subsequent integration into academic research, including laboratory studies and clinical validation.

Trigger	# of times reported	Existing scientific data	Ease of implementation	Score
Trigger 1	8	6	8	7.3
Trigger 2	6	6	6	6
Trigger 3	2	4	3	3
(1 = not reported, 10 = a lot reported)		(1 = no data, 10 = much data)	(1 = hard, 10 = easy)	

A darker colorization of the score indicated a higher prioritization.

### 4.2 Translating insights into preclinical and clinical research

After prioritization using the BKPM framework, the citizen-reported triggers are advanced through preclinical and clinical research stages designed to uncover mechanisms and test potential interventions. The first stage focuses on mechanistic exploration in preclinical human-based, vertebrate animal-free laboratory systems. Here, researchers study how triggers identified by citizens, such as stress hormones, histamine, environmental toxins, and genetic predispositions, affect atrial physiology. Hereto, experimental models including human induced pluripotent stem cell (iPSC)-derived atrial cardiomyocytes, HL-1 (immortalized mouse) atrial cardiomyocytes, and *Drosophila* AF are utilized and findings are validated in human atrial tissue and blood samples. As such, trigger-induced pathophysiological pathways (e.g., proteostasis, calcium handling, electrical conduction) are dissected how they potentially contribute to AF onset and progression. The mechanistic findings from this stage provide the biological foundation for developing targeted interventions.

The next stage involves validation and translation into clinical research, where hypotheses and mechanistic insights are tested in patients or advanced translational models. This phase includes several ongoing and completed clinical studies, such as the Glutaminimize trial (54), the GENIALITY trial (55), and the HF/AF-Energy trial (56) (Table 3). In addition, the establishment of the first familial AF outpatient clinic at Erasmus MC reflects the integration of citizen-informed knowledge into clinical care. Parallel investigations into histamine signaling, stress pathways, and environmental exposures such as pesticides exemplify how community-derived insights can guide both preclinical and clinical research. Many of these studies begin with mechanistic work in laboratory models and evolve into clinical testing once a plausible biological pathway has been established. Throughout these phases, bachelor’s and master’s students contribute to data analysis, experimental design, and science communication, reinforcing the educational mission of the CIRCULAR consortium and fostering new generations of researchers trained in participatory and translational science.

**Table 3:** Overview of past, ongoing, and future clinical trials and preclinical projects as part of project CIRCULAR (status October 2025).

Study/project	Focus	Approach	Status; outcomes
Glutaminimize trial	L-glutamine supplementation as a suppressor of AF	Clinical trial in patients with persistent AF and high HSP levels	Ongoing; early data suggest normalization of energy metabolites and improved atrial function based on (54)
Familial AF	Young AF patients without risk factors	Whole Genome Sequencing	Gene variants that may trigger AF
AF-Energy trial	NAD (nicotinamide riboside) to restore NAD + metabolism	Clinical trial in AF with heart failure	Ongoing; based on preclinical protection against DNA damage and mitochondrial dysfunction (58, 73)
Geniality trial	HSP inducing compound GGA to prevent POAF	Clinical trial in patients undergoing cardiac surgery, and detection of new onset AF post-surgery	Ongoing; based on (pre)clinical data showing GGA to protect against AF (61, 62)
Plant-based diet intervention	Whole-food, plant-based diet for AF suppression	Lifestyle trial co-designed with citizens	Planned; initial surveys confirm positive effects on HRQoL (63)
Stress and AF	Role of psychological stress and stress proteins on AF burden	Cohort studies and biomarker analysis	Ongoing
Pesticide exposure study	Environmental triggers of AF	Biomonitoring (blood/tissue pesticide levels) correlated with AF biobanks	Ongoing; collaboration with non-profit Meten = Weten and SPRINT project (74)
Human AF Electropathology Atlas	Mapping atrial bio-electrical remodeling across clinical AF stages	Multi-omics analyses: transcriptomics, proteomics and metabolomics, electrical mapping	In progress; obtaining omics insights combined with electrical signals (75, 76)
AF-on-a-Chip platform	High-throughput testing of drugs, toxins, and lifestyle-related molecules	Microfluidics with human cardiomyocytes	In development; will support scalable screening
Animal-free AF models	Mechanistic studies of AF triggers and suppressors	iPSC-derived atrial cardiomyocytes, HL-1 cells, Drosophila	Active; revealed effects of pesticides, stress, histamine, and genetic mutations

**Figure 4:**
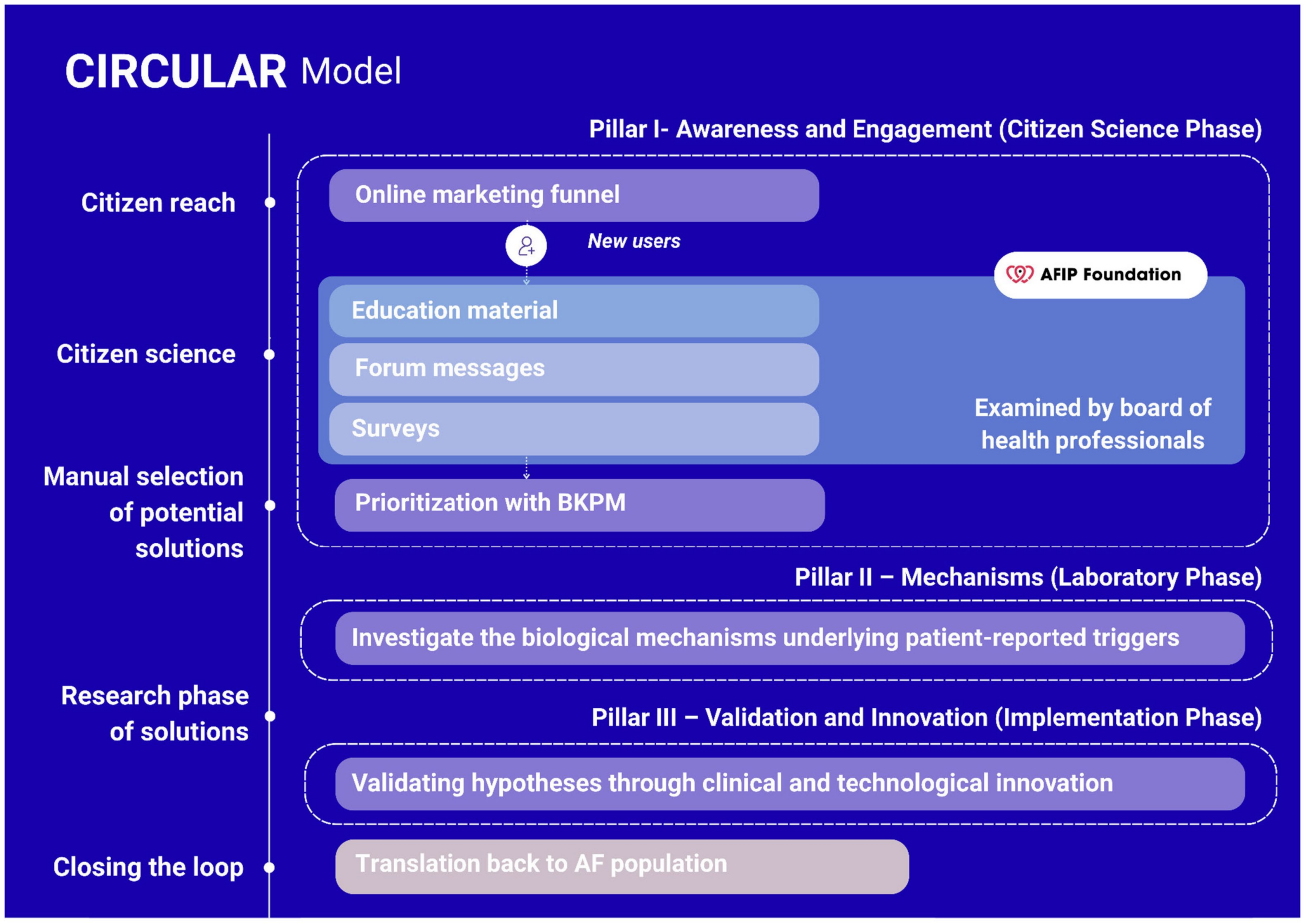
The three-pillar structure of the CIRCULAR project. This figure illustrates the integrated research and education framework of the CIRCULAR consortium. Pillar I (Awareness and Engagement) represents the CS phase, where individuals with AF share lived experiences, generate hypotheses, and contribute to participatory research and learning. Pillar II (Mechanisms) translates these insights into mechanistic understanding using human-based, animal-free laboratory models, engaging students and early-career researchers in advanced experimental training. Pillar III (Validation and Innovation) focuses on testing and validating interventions and technologies inspired by citizen insights, combining translational research with educational activities in innovation and communication. Together, the three pillars form a circular feedback system in which experiential knowledge drives scientific discovery, and results are shared back with the AF community to foster transparency, education, and continued engagement.

The CIRCULAR project places citizens with lived experiences on AF at the core of the research process. Insights on potential triggers and suppressors of AF are systematically gathered through surveys, online discussions, and outreach campaigns, and subsequently evaluated by health professionals and researchers. Each reported factor is assessed for its frequency, existing scientific evidence, and feasibility for investigation in experimental AF models (Table 2). These insights serve as the foundation for mechanistic laboratory studies or clinical validation, thereby connecting experiential knowledge with biomedical investigation. This approach has already revealed several contributors to AF onset and progression, including psychological stress, genetic predisposition, environmental pesticide exposure, allergies, and diet (Table 3). To translate these experiences into scientific evidence, the consortium is organized into three interconnected pillars, each representing a phase in the circular research process (Figure 4).

#### 4.2.1 Pillar I—awareness and engagement (citizen science phase)

This pillar is where CS is most visibly implemented. It focuses on collecting, analyzing, and validating citizen-reported triggers and suppressors of AF through online platforms, surveys, and outreach campaigns. Participants contribute their lived experiences and propose hypotheses, which are then prioritized by researcher using the BKPM framework. These insights are subsequently linked to cohort studies, genetic analyses, and biobanks such as AFFIP (57). In addition to engaging citizens, this pillar also has a strong educational dimension. Students at the bachelor’s and master’s levels participate in data collection, analysis, and communication activities, together with people living with AF, hereby learning how to integrate experiential knowledge into scientific inquiry. Educational modules and research internships within the CIRCULAR consortium introduce students to the principles of participatory research, health literacy, and co-creation, fostering mutual learning between citizens, researchers, and future health professionals.

#### 4.2.2 Pillar II—mechanisms (laboratory phase, citizen-informed)

This pillar represents the pre-clinical follow-up to citizen-generated hypotheses. While it does not involve direct citizen participation, it remains citizen-informed, as it translates community insights into mechanistic understanding. Researchers investigate how identified triggers affect atrial physiology using human-based, vertebrate animal-free models, including iPSC-derived atrial cardiomyocytes, HL-1 cardiomyocytes, *Drosophila,* and human atrial tissue and blood samples (58, 59). These studies aim to elucidate how stress hormones, histamine, environmental toxins, wear-and-tear, and genetic predispositions alter proteostasis, calcium handling, and electrical conduction, leading to AF. This pillar also contributes to education and training within the consortium. Students and early-career scientists are actively involved in experimental design, bioinformatics, and mechanistic interpretation, learning advanced techniques in molecular cardiology and translational biology. The work is aimed to culminate in the development of the Human AF Electropathology Atlas, an open-access database integrating structural, electrophysiological, and molecular data. By making this knowledge publicly available, the consortium maintains indirect engagement with the AF community while promoting scientific literacy among students and professionals.

#### 4.2.3 Pillar III—testing and innovation (translational phase, citizen-informed)

Pillar III focuses on testing and validating interventions and technologies derived from earlier pillars. Although this phase is not CS in the participatory sense, it remains citizen-informed, as it operationalizes hypotheses rooted in patient experiences. The pillar evaluates therapeutic and preventive strategies such as dietary programs, nutraceuticals [e.g., L-glutamine (54), nicotinamide (58, 60)], antihistamines, Heat Shock Protein inducer (61, 62), and other lifestyle-based interventions (63). In parallel, technological innovations such as the AF-on-a-Chip platform allow high-throughput testing of compounds, toxins, and patient-specific treatments before clinical implementation. An educational dimension is also embedded here. Students and trainees gain experience in translational research, ethical innovation, and regulatory science, learning how to bridge laboratory findings with real-world application. Through involvement in validation studies and dissemination activities, they develop skills in cross-disciplinary teamwork and communication with both scientific and public audiences. Results from this pillar are shared with the AFIP community, ensuring transparency and completing the circular feedback loop to Pillar I.

Together, the three pillars form a circular and iterative research pipeline that connects citizen input, laboratory discovery, and clinical application while embedding education throughout (Figure 4). Direct participation by citizens occurs primarily in the first phase, where lived experiences generate hypotheses and guide research priorities. The subsequent phases translate these hypotheses into mechanistic studies and test them through experimental and clinical validation, involving students and early-career researchers in each step. Through this integration of participatory and educational activities, citizens, students, and scientists engage in a continuous process of mutual learning and knowledge exchange. This design ensures that citizens meaningfully shape the research agenda, while education strengthens long-term capacity for participatory and translational science. In doing so, CIRCULAR exemplifies how CS can drive biomedical discovery and innovation while upholding scientific rigor, ethical standards, and societal relevance.

### 4.3 Citizen science in education

CS is increasingly recognized as a powerful educational approach that fosters scientific literacy, engagement, and empowerment among learners. By involving students and citizens in authentic research, through data collection, question formulation, or interpretation of results, CS transforms traditional education into an active, inquiry-driven process (64, 65). Integrating CS into formal and informal educational contexts has been shown to improve understanding of the scientific process, increase motivation, and strengthen civic responsibility (66). Participants gain not only technical and analytical skills but also an appreciation of how science contributes to addressing real-world challenges. In the context of health education, CS is particularly valuable for enhancing health literacy and promoting self-efficacy. By engaging citizens and students in projects related to (chronic) disease prevention, lifestyle modification, and environmental health, CS helps individuals to understand the links between personal behavior, community wellbeing, and scientific evidence (67). This participatory approach not only empowers citizens to make informed health decisions but also engages students as active learners who draw on citizens’ experiential knowledge, thereby linking academic education with practical insights from daily life.

Within this framework, the CIRCULAR project provides a pioneering example of how CS can be integrated into education and biomedical research. In addition to its scientific objectives, CIRCULAR includes dedicated educational components in which bachelor’s and master’s students contribute to CS activities. Students analyze citizen-reported data on AF triggers and suppressors, participate in laboratory experiments testing these insights, and help design communication strategies to explain CS outcomes and engage broader audiences. These activities expose students to the full research cycle, from hypothesis generation based on lived experience to experimental validation, and highlight the societal relevance of co-created science. By embedding CS into (higher) education and health communication, CIRCULAR not only generates new scientific knowledge but also cultivates a new generation of researchers and professionals skilled in participatory and transdisciplinary collaboration. This integration of education, citizen engagement, and biomedical research demonstrates the broader potential of CS to bridge gaps between science, society, and public health.

## 5 Expanding citizen science beyond atrial fibrillation

The CS model developed within project CIRCULAR and implemented via the AFIP foundation demonstrates how lived experience of citizens with AF can be integrated with biomedical, clinical and public health research and education to generate testable hypotheses and strengthen community engagement. This approach aligns with the ECSA Ten Principles, which emphasize meaningful roles for citizens (contributors, collaborators, or leaders) and open, reciprocal communication, principles that are portable across disease domains (68).

### 5.1 A scalable framework for chronic disease research

CIRCULAR’s three-phase structure, citizen awareness and engagement (Pillar I), mechanistic investigation (Pillar II), and translational testing (Pillar III), can be adapted to other chronic and complex conditions (e.g., diabetes, heart failure, COPD, neurodegeneration), where lifestyle, environmental, and psychosocial factors interact with biomedical mechanisms. Evidence from patient and public involvement shows that participation improves the relevance, quality, and legitimacy of research and its implementation, key arguments for scaling CS to additional disease areas (69). And even though CS activities from CIRCULAR cannot be classified as participatory level A, as defined by Den Broeder (13), and only partially falls into level B of Den Broeder’s framework, CIRCULAR’s approach can be the basis of future research integrating CS with biomedical methods.

### 5.2 Building digital and educational capacity

AFIP’s digital ecosystem (blogs, forums, social media, newsletters) can evolve into an open CS hub hosting multiple disease-specific subcommunities while sharing governance, data standards, and good practice. Public health perspectives on CS highlight its roles in agenda setting, co-creation, and capacity building, supporting both scientific and civic learning (70). Educational integration is equally important: embedding CS in coursework and supervised projects develops a workforce fluent in participatory, transdisciplinary methods—an approach consistent with contemporary CS education research and guidance (68).

### 5.3 Partnerships and ethical data integration

Scaling beyond AF requires partnerships among research institutes, healthcare systems, citizen organizations, and technology companies, coupled with interoperable data infrastructures (e.g., secure data donation, wearables, environmental mapping) (71). In parallel, ethical data governance frameworks for biomedical CS, covering ownership, reciprocity, and risk–benefit assessment, provide practical guidance to ensure citizen control and trust at scale (72).

### 5.4 Broader public health impact

Extending CS across chronic diseases can advance equity, prevention, and patient empowerment, uncovering shared determinants (e.g., stress, pollution, diet) and informing population-level strategies. Public health scholarship increasingly recognizes CS as a lever for health equity and policy-relevant evidence, reinforcing its relevance beyond single-disease programs (72). By adhering to open access, reciprocity, and co-creation, the same principles that guided CIRCULAR, this framework can evolve from a disease-specific initiative into a societal model for participatory public health research, linking citizens, clinicians, and scientists in the collective pursuit of healthier lives (68).

### 5.5 Transferable principles and context-specific elements

While the CIRCULAR model is rooted in the specific context of AF research in the Netherlands, it comprises both transferable principles and context-dependent elements. Transferable aspects include the circular research logic linking citizen-derived experiential knowledge to hypothesis generation, prioritization, mechanistic investigation, and clinical validation thereby extending CS into preclinical and mechanistic biomedical research. In addition, using engagement and feedback infrastructures to sustain long-term participation and reciprocal learning. These elements are disease-agnostic and can be adapted to other chronic and multifactorial conditions where lived experience intersects with biological mechanisms. At the same time, several components could be seen as more context-specific. These include the characteristics of AF as an episodic, symptom-driven condition that encourages active self-observation by patients. Also, the existence of a long-standing and trusted OHC through the AFIP Foundation and also features of the Dutch research, funding, and governance landscape that enabled close integration of CS with biomedical research. Recognizing this distinction is essential for responsible scaling, as successful transfer to other diseases or settings requires adaptation and context-reflexivity rather than direct replication.

## 6 Limitations and challenges

While the CIRCULAR model illustrates the potential of CS to connect lived experience with biomedical and public health research, several limitations warrant consideration. First, OHC are subject to representation bias, as participation typically favors individuals with higher digital literacy, education, and access to online platforms. Consequently, certain populations may be underrepresented, and citizen-reported insights should not be assumed to be fully representative of all people living with AF. Second, CS approaches carry a risk of over-prioritizing frequently reported triggers or suppressors, potentially overlooking clinically rare but severe mechanisms. Within CIRCULAR, this risk is partly mitigated through the BKPM, which combines reporting frequency with existing scientific evidence and feasibility of investigation. Nevertheless, prioritization remains an inherently normative process, and complementary strategies are required to ensure that high-impact but less frequently reported phenomena receive adequate attention. Third, power asymmetries persist even within structured participatory frameworks. Although citizen input plays a central role in hypothesis generation, decisions regarding translation into preclinical and clinical research are ultimately shaped by researchers, institutional constraints, and funding priorities. As such, CS in biomedical contexts often operates along a continuum from citizen-generated to citizen-informed research rather than fully citizen-led inquiry. Implementation of citizen science models such as CIRCULAR is inherently iterative and requires ongoing adaptation in response to emerging challenges. While early outcomes indicate that the current model is effective, these findings do not preclude the need for continued refinement as the project evolves. Future work and reporting will therefore explicitly incorporate reflexive evaluation to ensure that the model remains responsive, robust, and contextually appropriate over time. Finally, there are ethical and epistemic tensions in translating anecdotal experiential insights into biomedical experimentation. Lived experience can generate valuable hypotheses but does not constitute evidence in the traditional biomedical paradigm. To address this, CIRCULAR treats citizen-derived insights as starting points for inquiry that require rigorous validation through mechanistic studies and ethically approved clinical research, thereby safeguarding scientific rigor and participant trust.

## 7 Conclusion: the CIRCULAR model of co-creation as a framework for citizen-driven biomedical innovation

The CIRCULAR model demonstrates how CS can be integrated into biomedical and public health research through structured co-creation. By using lived experience as a starting point for hypothesis generation and linking it to mechanistic investigation and clinical translation, CIRCULAR connects experiential knowledge with the biomedical research pipeline in a reciprocal and iterative manner. Three transferable insights emerge. First, citizen-derived experiential knowledge can be scientifically generative, producing hypotheses that may not arise through conventional research pathways. Second, sustained engagement of all stakeholders relies on reciprocity, with transparency and feedback strengthening long-term participation. Third, integration across research stages, from experiential observations to mechanistic and clinical testing, is essential for translating lived experience into actionable biomedical and public health innovation. Although developed in the context of AF, these principles are applicable to other complex chronic diseases where lived experience intersects with biological mechanisms, offering a pathway toward more inclusive and socially relevant health research. Therefore, CIRCULAR can serve as an inspiration for implementations of CS in the biomedical research ecosystem.
